# Prevalence of developmental anomalies in teeth among Indian school children: An epidemiological study

**DOI:** 10.6026/973206300211135

**Published:** 2025-05-31

**Authors:** Monisha Batra, Awadhesh Gupta, Savita Singh, Salma F., Rahul Kumar Tomar, Sambit Prasad, Yatharth Yatharth

**Affiliations:** 1 Private Clinical Practitioner, Chadakya, New Delhi, India; 2 Department of Oral & Maxillofacial Pathology, D. J. College of Dental Sciences & Research, Modinagar, Ghaziabad (U.P), India; 3 Self Owned Multispecialty Hospital & Dental Clinic, Chirag Delhi - New Delhi, India; 4 Department of Oral & Maxillofacial Pathology, NIMS Univeraity, Jaipur, Rajasthan, India; 5 Department of Oral Pathology and Microbiology, DJ College of Dental Sciences and Research, Modinagar, Ghaziabad, Uttar Pradesh, India; 6 Department of Dental Surgeon, Community Health Centre, Odagaon, Nayagarh, Odisha, India; 7 Intern, Kalinga Institute of Dental Sciences, Kalinga Institute of Industrial Technology (KIIT), Deemed to be University, Patia, Bhubaneswar, Odisha, India

**Keywords:** Developmental anomalies, prevalence, epidemiological study, enamel hypoplasia, supernumerary teeth, fusion, microdontia, hypodontia, dens evaginatus, talon cusp

## Abstract

The prevalence of developmental dental anomalies among 5,000 school children aged 5-15 years in Ghaziabad, Uttar Pradesh is of interest. We examined their association with age, sex, dentition stage, jaw location, and socioeconomic status. Data were collected from school-based screening camps and pediatric outpatients at Santosh Dental College. Enamel hypoplasia was the most common anomaly, followed by supernumerary teeth and talon cusp, while micro-dontia, fusion, and hypodontia were less frequent. Most anomalies showed no gender bias, except for enamel hypoplasia and talon cusp. Higher prevalence was observed in children from lower socioeconomic groups, in the mixed dentition stage, and predominantly in the maxilla.

## Background:

Developmental dental anomalies originate prenatally, manifesting either at birth or later, shaped by both genetic and environmental influences [1]. These anomalies disrupt normal tooth number, form, size, structure, and development, encompassing conditions such as supernumerary teeth, hypodontia, enamel hypoplasia, amelogenesis imperfecta, and fusion [1, 2]. They arise during the complex stages of tooth morphogenesis-bud, cap, and bell-governed by intricate epithelial-mesenchymal interactions and tightly regulated genetic signalling pathways [2, 3]. Therefore, it is of interest to evaluate the occurrence and associated factors of dental anomalies in school-going children of Ghaziabad.

## Methodology:

A cross-sectional epidemiological investigation was undertaken among a cohort of 5,000 school-aged children, ranging from 5 to 15 years, residing in the Ghaziabad district of Western Uttar Pradesh. The study population was obtained through a stratified random sampling approach, encompassing participants from school-based oral health outreach programs as well as pediatric patients attending a tertiary dental care facility. Prior to commencement, ethical clearance was secured from the institutional review board, and formal authorization was granted by respective school administrations. Informed written consent was obtained from the parents or legal guardians, and pertinent socio-demographic information was elicited via a pre-validated structured questionnaire. Eligibility criteria included children within the specified age range presenting with developmental dental anomalies concerning tooth number, morphology, dimensions, and structural integrity. Individuals with systemic pathologies known to influence odontogenesis or those falling outside the age criteria were excluded from the study. Clinical examinations were conducted onsite within school settings under ambient natural illumination, utilizing sterilized diagnostic instruments including mouth mirrors and dental explorers. High-resolution intraoral photographs were captured to facilitate anomaly documentation. The dental anomalies assessed encompassed supernumerary dentition, hypodontia, microdontia, macrodontia, talon cusp, dens evaginatus, peg-shaped maxillary lateral incisors, dental fusion, and enamel hypoplasia. Talon cusps were systematically classified according to the typology delineated by Hattab *et al.* (1996) [3] as follows:

[1] Type I (True Talon): A prominently defined cusp projecting from the palatal aspect, extending ≥50% of the distance from the cementoenamel junction (CEJ) to the incisal margin.

[2] Type II (Semi talon): A less pronounced projection extending <50% of the CEJ-incisal edge distance, either fused with or distinct from the palatal surface.

[3] Type III (Trace Talon): Mildly hypertrophic cingulum presenting as conical, bifid, or tuberculate formations.

All data were systematically entered and subjected to statistical analysis using SPSS software version 21.0 and Epi-info version 3.0. The Chi-square test was applied to explore associations between categorical variables. A significance threshold of p < 0.05 was adopted, with confidence intervals computed at the 95% level. Children identified with developmental anomalies were duly counselled and referred for further dental intervention as warranted.

## Results:

In the present study school children aged between 5-15 years were surveyed for the developmental dental anomalies. Out of the total 5,000 children examined, 2,661 (53.22%) were males, and 2,339 (46.78%) were females. The total number of affected cases was 128, accounting for 2.56% of the population. Among the anomalies, enamel hypoplasia was the most frequently observed condition (1.2%), followed by supernumerary teeth (0.4%), peg-shaped laterals (0.28%), and talon cusp (0.26%). Less common anomalies included fusion (0.24%), hypodontia (0.12%), and microdontia (0.1%).The overall prevalence was comparable between males (2.59%) and females (2.5%). However, the occurrence of enamel hypoplasia was significantly higher in males (1.42%) than females (0.9%) (p = 0.039). Conversely, talon cusp was more prevalent among females (0.4%) compared to males (0.2%) (p = 0.049). The predominant cause of enamel hypoplasia among the 58 affected children was fluorosis, accounting for 56.89% of cases. This was followed by nutritional deficiencies, contributing to 27.58% of the cases, likely linked to the low socioeconomic status of the families. Other less common factors included exanthomatous diseases (6.8%), low birth weight (5.1%), and birth injuries (3.44%) (Table 1). The prevalence of enamel hypoplasia and talon cusp showed statistically significant variation across different socioeconomic groups (p < 0.05). Enamel hypoplasia was most common in Group B (income 10,000-20,000), while talon cusp prevalence was also higher in the same group (p > 0.05) (Table 2). The statistical analysis confirms the absence of significant associations between age and the occurrence of these dental anomalies (all p-values > 0.05).Overall, the maxilla is more frequently affected, accounting for 56.9% of cases, compared to 43% in the mandible. Specific anomalies such as talon cusp (69.2%) and peg-shaped laterals (71.4%) show a pronounced predilection for the maxilla. In contrast, fusion anomalies are more common in the mandible (58.3%), while hypodontia and supernumerary teeth are equally distributed between both arches. The majority of cases were observed in the mixed dentition group (ages 6-12 years), comprising 106 of the total 128 anomalies. Enamel hypoplasia was the most common anomaly overall (45.3%), predominantly affecting mixed dentition (44.3%). Supernumerary teeth (15.6%) and peg-shaped laterals (10.9%) were also primarily seen in the mixed dentition phase. Talon cusps and fusion showed a lower but consistent presence across all dentition types. Primary dentition (age 5) had fewer cases, with anomalies such as enamel hypoplasia and fusion each representing 14.28% of their respective totals in this group. Permanent dentition (ages 13-15) had the least involvement. The majority of cases were equally divided between True Talon (38.46%) and Semi Talon (38.46%), with Trace Talon accounting for 23% of cases.

## Discussion:

Among 5000 schoolchildren surveyed, 2.56% exhibited developmental dental anomalies. The cohort included 2339 females (46.8%) and 2661 males (53.2%). Observed anomalies included enamel hypoplasia, supernumerary teeth, talon cusp, microdontia (peg-shaped laterals), hypodontia, and fusion. Enamel hypoplasia-characterized by defective enamel matrix formation-was detected in 1.2% of the population. This prevalence is lower than that reported by Nayak *et al.* in India (18.8%) [4], yet higher than the findings of Uslu *et al.* in Turkey (0.4%) [5]. The variation suggests a multifactorial etiology influenced by genetic predisposition and environmental conditions. In our study, developmental enamel defects were more prevalent in males (1.42%) than females (0.9%), aligning with findings by Dummer *et al.* (1986) [6]. However, Lunardelli *et al.* (2005) [7] reported a higher prevalence among females, highlighting potential gender-based variation.In the present study, 57% of enamel hypoplasia cases were attributed to fluorosis, with 15.1% exhibiting surface pits-exceeding the incidence noted by Dummer *et al.* (1986) [6]. While fluorosis prevalence in Uttar Pradesh, predominantly affecting males as per our finding's highest occurrence in the 6-12-year age group was noted. Of the 36 children identified with enamel hypoplasia from low socioeconomic group, presented with 44.4% showing signs of nutritional deficiency. This observation supports findings by Niswander *et al.* [8], who reported a greater occurrence of enamel hypoplasia among malnourished children in both Japanese and Indian populations. Previous studies have linked low birth weight to enamel hypoplasia; however, only 5% of children in our study reported low birth weight, significantly lower than the 62.3% reported by Seow *et al.* [9], likely due to differing systemic and local influences. Enamel hypoplasia in this study was linked to birth injury (3.44%) and prolonged fever from exanthematous diseases (6.8%), supporting Seow *et al.*'s findings that systemic factors like birth trauma, fever, and nutritional deficiencies contribute to its development. In our study, supernumerary teeth were observed in 0.4% of subjects, aligning with Jarvinen *et al.*'s findings [10]. Supernumerary teeth most commonly occurred in mixed dentition in our study, reflecting varied prevalence rates across populations with clinical implications for timely diagnosis and management [11]. The 0.4% prevalence of supernumerary teeth in Western UP, predominantly in the anterior maxilla (50%), aligns with previous findings and underscores the clinical importance of early detection due to its developmental origin and potential complications [12].

Niswander and Sujaku [8] proposed a genetic basis for supernumerary teeth with an X-linked autosomal dominant pattern affecting females, reflected in our study where 20% reported family history-10% in siblings and 10% in parents-emphasizing its clinical significance for early diagnosis and risk prediction. Our study reported a talon cusp prevalence of 0.26%, lower than Guttal *et al.*'s 4.28% [13]. Consistent with literature, talon cusp was rare in deciduous teeth, with 11 of 13 cases observed in mixed dentition on permanent teeth. Following Hattab *et al.*'s [3] classification, true and semi talon types predominated, primarily affecting maxillary lateral incisors. Our study's findings align with Uslu *et al.* [5] confirming the low prevalence of microdontia. Our study found microdontia more common in females, contrasting with Buenviaje *et al.* [14], who reported higher prevalence in males. With a prevalence of 0.28%, peg-shaped laterals ranked third in our study, showing higher female occurrence, unlike Nayak *et al.* [4] who found no gender difference; notably, our cases were mainly in mixed dentition, contrasting Ooshima *et al.* [15] who reported higher prevalence in primary dentition. Our study reported 0.1% hypodontia-lower than Warnakulasuriya [16] with equal jaw involvement and higher prevalence in mixed dentition, differing from earlier findings [17]. Familial history in 2 of 6 cases supports genetic links (Shimizu & Maeda) [18]. Fusion (0.24%) matched mostly in mixed dentition and low socioeconomic groups, indicating nutritional influence; gender trends aligned with Guttal *et al.* [13] (male predominance) though Salem noted female bias. Early diagnosis of dental anomalies enables timely intervention, improving functional and aesthetic outcomes in pediatric patients. The study's regional focus and sample size may limit the generalizability of the findings. Larger, multicentric studies are needed to explore genetic and environmental factors influencing dental anomalies.

## Conclusion:

A 2.56% prevalence of developmental dental anomalies in Western Uttar Pradesh, with enamel hypoplasia being most common and a higher incidence in low socio-economic, mixed dentition children, predominantly affecting the maxilla is seen. Most anomalies showed no gender bias, except for enamel hypoplasia and talon cusp. Higher prevalence was observed in children from lower socioeconomic groups, in the mixed dentition stage, and predominantly in the maxilla.

## Figures and Tables

**Table 1:** Etiological factors causing enamel hypoplasia

**Etiological Factor**	**Number of Cases (n=58)**	**Percentage (%)**
Fluorosis	33	56.89
Nutritional Deficiency	16	27.58
Exanthomatous Diseases	4	6.8
Low Birth Weight	3	5.1
Birth Injury	2	3.44

**Table 2:** Dental anomalies by socioeconomic status

**Dental Anomaly**	**Group A (<10,000)**	**Group B (10,000-20,000)**	**Group C (20,000-30,000)**	**Group D (>30,000)**	**p-value**
Enamel hypoplasia	10 (1.01%)	26 (1.74%)	17 (0.76%)	5 (1.7%)	0.038*
Talon cusp	2 (0.2%)	9 (0.6%)	2 (0.08%)	0 (0.0%)	0.017*
Supernumerary teeth	3 (0.3%)	10 (0.66%)	6 (0.2%)	1 (0.3%)	0.269
Peg shaped laterals	2 (0.2%)	7 (0.46%)	5 (0.22%)	0 (0.0%)	0.365
Fusion	3 (0.3%)	7 (0.46%)	2 (0.08%)	0 (0.0%)	0.1
Hypodontia	0 (0.0%)	4 (0.6%)	2 (0.08%)	0 (0.0%)	0.219
Microdontia	1 (0.1%)	3 (0.2%)	1 (0.04%)	0 (0.0%)	0.476
